# Authors’ reply to the comment from Uchida et al.

**DOI:** 10.1186/s13054-023-04606-3

**Published:** 2023-08-25

**Authors:** Itsuki Osawa, Kent Doi

**Affiliations:** Department of Emergency and Critical Care Medicine, The University of Tokyo Hospital, 7-3-1, Hongo, Bunkyo-Ku, Tokyo, 1130033 Japan

We thank Uchida and Hayashi for their comments on our study [1]. As pointed out by Uchida et al. [2], we acknowledge the potential survivor treatment selection bias resulting from the use of data from a retrospective observational study (the JSEPTIC-DIC study) to identify the best criteria for polymyxin B hemadsorption (PMX-HA). However, because the derived criteria were validated in the analysis using data from the EUPHRATES trial, in which treatment allocation is randomized, we believe that the impact of selection bias was well minimized.

To address the potential immortal time bias [3], we analyzed only those cases that were alive for at least 54 h after randomization (i.e., the maximum time from randomization to the end of the standard regimen of two PMX-HA regimens) [4, 5]. As shown in Additional file 1: Fig. S1, the hazard ratios up to 28 days using the Cox proportional hazard model, adjusted for baseline APACHE II and SOFA scores, are 0.79 (95% CI 0.53–1.19, *p *= 0.26) and 0.64 (95% CI 0.41–0.99, *p *= 0.04) in all and targeted population, respectively. Therefore, we believe that these results from the analysis considering immortal time remained consistent with the original findings. However, it should be noted that the results of this additional analysis may underestimate the effect of PMX-HA by assuming that early deaths in the control group that were excluded from the analysis considering immortal time, could have been saved if they had been assigned to the PMX-HA group.

Although the two potential biases indicated by Uchida et al. could pose limitations to our study, they do not significantly undermine the target subpopulation of PMX-HA when considering the results of our additional analysis. However, the validity of our findings needs to be verified in future prospective studies.

### Supplementary Information

**Additional file 1: Fig. S1.** Kaplan-Meier curves of patients in all and targeted population of the validation cohort considering immortal time.

## Data Availability

The data from the JSEPTIC-DIC study are publicly available (*Sci Data.* 2018;5:180243). The data from the EUPHRATES trial will not be shared beyond the requests from investigators of its trial.
