# Supplementary material for: Authors’ reply to the comment from Uchida et al
Source: Crit Care. 2023 Aug 25;27:327. doi: 10.1186/s13054-023-04606-3 (PMC10464252; doi:10.1186/s13054-023-04606-3)
Supplement: Supplementary file 1 — Additional file 1: Fig. S1. Kaplan-Meier curves of patients in all and targeted population of the validation cohort considering immortal time. [file 13054_2023_4606_MOESM1_ESM.docx]

# **Supplemental Figure 1. Kaplan-Meier curves of patients in all and targeted population of the validation cohort considering immortal time.**


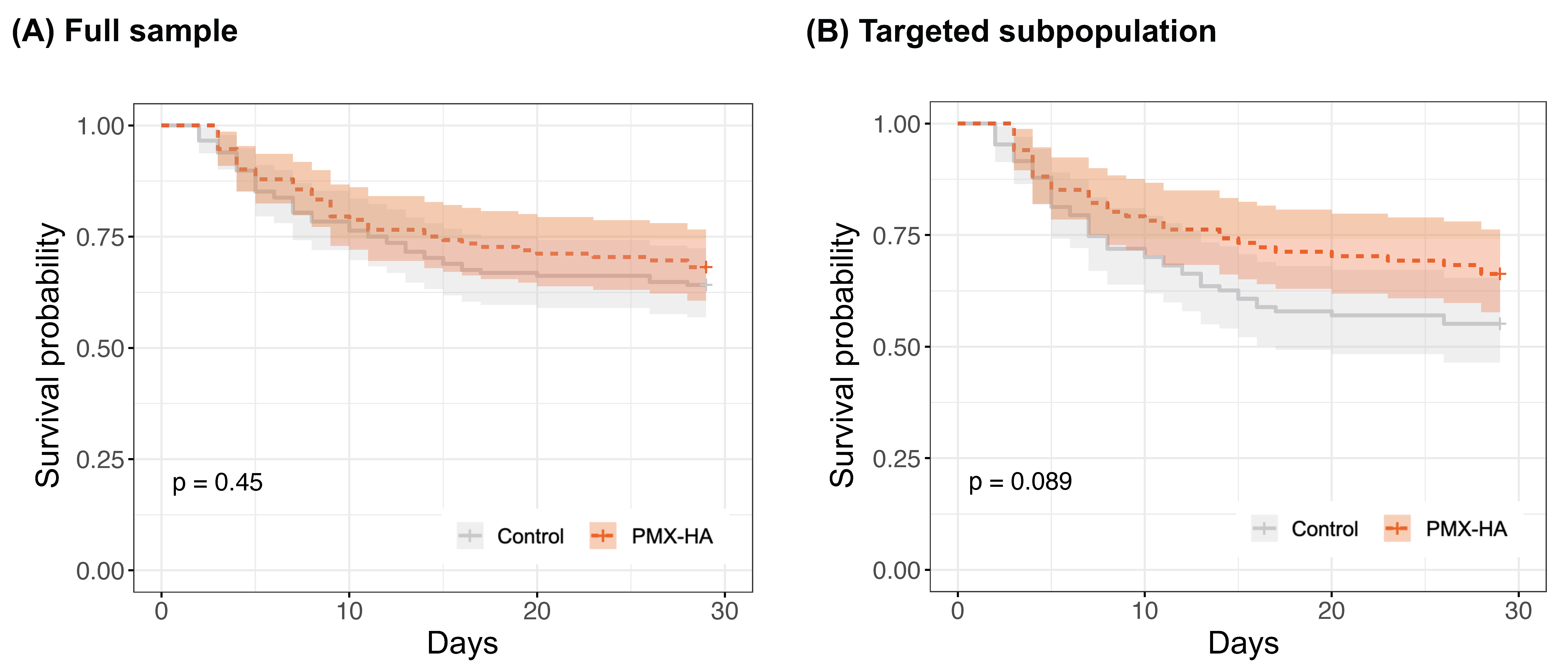


Shown are unadjusted Kapan-Meier curves of patients in (A) all and (B) targeted population of the validation cohort. “Targeted subpopulation” indicates patients with PT-INR > 1.4 or lactate > 3 mmol/L on ICU admission. P values were estimated by the log-rank test. To address the potential immortal time bias, we analyzed only those cases that were alive for at least 54 hours after randomization (i.e., the maximum time from randomization to the end of the standard regimen of two PMX-HA regimens). The hazard ratios up to 28 days using the Cox proportional hazard model, adjusted for baseline APACHE II and SOFA scores, are 0.79 (95% CI [0.53 to 1.19], p = 0.26) and 0.64 (95% CI [0.41 to 0.99], p = 0.04) in all and targeted population, respectively.

Abbreviations: PMX-HA = Polymyxin B Hemadsorption, PT-INR = Prothrombin Time and International Normalized Ratio, APACHE II = Acute Physiology And Chronic Health Evaluation II, SOFA = Sequential Organ Failure Assessment
